# Monogenic etiologies in a cohort of early onset obesity: a real-world experience from Belgium

**DOI:** 10.3389/fendo.2025.1608398

**Published:** 2025-08-01

**Authors:** Julie Harvengt, Muriel Hannon, Leonor Palmeira, Marie-Christine Lebrethon, Vinciane Dideberg, Vincent Bours

**Affiliations:** ^1^ Human Genetics Department, CHU of Liège, Liège, Belgium; ^2^ GIGA Research, University of Liège, Liège, Belgium; ^3^ Pediatric Endocrinology, Pediatric Department, CHU of Liège, Liège, Belgium

**Keywords:** early-onset obesity, monogenic obesity, MC4R, Bardet–Biedl syndrome, hypothalamic obesity

## Abstract

**Introduction:**

Obesity is a major global health issue with multifactorial etiologies. Among them, recent advances in the comprehension of eating and energy regulation showed that around 60 genes involved in the hypothalamic leptin/melanocortin pathway contribute to the development of rare monogenic or syndromic forms of obesity.

**Objective:**

To better delineate the genetic diagnostic rate and the phenotype in a cohort of early onset obesity and to integrate our results in guidance for genetic testing.

**Methods:**

In a diagnostic setting, 223 patients with early onset obesity were screened through a targeted panel including 44 genes for severe early onset obesity. Genetic results and clinical descriptions were reviewed for the entire cohort.

**Results:**

A diagnostic yield of 3.1% was established. Likely pathogenic or pathogenic variants were found in *MRAP2*, *MC4R*, *BBS2*, and *BBS4*, and a 16p11.2 deletion was confirmed. Clinically, 23% of the cohort had early onset obesity at <1 year, 47% at 1–4 years, and 30% at >4 years. No discriminative clinical feature appears to enhance the diagnostic yield. Thirty-six percent of the cohort presented additional neurological complaints that led to more extensive genetic investigations with a diagnosis rate of 1.8% in this subgroup.

**Conclusion:**

Our work found a diagnostic yield of 3.1%. Additionally, 19.7% of heterozygous variants of unknown significance were found in genes related to autosomal conditions and 34.9% in genes related to recessive conditions. These results highlight the need for accurate genotype-phenotype correlations. Genetic laboratory expertise in obesity is highly recommended, especially in the context of the availability of new targeted anti-obesity therapies that open the field for current and future perspectives of these targeted genetic investigations.

## Introduction

Childhood obesity has been recognized as one of the most serious public health problems of the 21st century. In Belgium, recent epidemiological studies (1) reveal that nearly half (49%–55%) of the adult population aged more than 18 years is overweight (BMI ≥ 25) and 16% is obese (BMI ≥ 30). In the group of children and adolescents (2–17 years), 19% present an excess weight (85th percentile ≤ BMI < 95th percentile), and 5.8% are obese (BMI ≥ 95th percentile). A standard pediatric categorization defines obesity in three classes by using the 95th percentile for age and sex as the reference threshold and categorizing 100%–120% of the 95th percentile as class I obesity, 120%–140% as class II obesity, and more than 140% as class III obesity (2, 3). Among the children presenting severe obesity (i.e., classes II and III obesity), at least 5%–10% present chromosomal abnormalities and/or highly penetrant genetic mutations that contribute to their obesity (4).Individually, these monogenic disorders are considered (very) rare. But at a population level, the impact of these diagnoses may be significant on public health care. Moreover, the current medical practices tend to be more oriented to a better precision medicine that might be started at younger ages for children accurately diagnosed.

Clinicians face the challenge of identifying the rare genetic forms of obesity among the large population group of severely obese young children and adolescents. Until now, one major criterion has been to start genetic investigations in cases of inappropriate weight gain in comparison to diet and lifestyle. As a main symptom, sustained severe hyperphagia (moreover with nocturnal eating) from early childhood is a feature of the genetic obesity syndromes (5, 6). Learning and behavioral troubles, developmental delay (e.g., Prader–Willi syndrome), ophthalmological issues and/or kidney failure (e.g., visual loss/renal abnormalities encountered in Bardet–Biedl syndrome (7)) are classically encountered in these syndromes, leading generally to an exhaustive genetic work-up at a very young age. However, over the last 20 years, a group of genetic disorders with severe obesity as the only presenting feature has emerged. These monogenic non-syndromic obesity disorders are mainly driven by molecular alterations in hypothalamic pathways involved in appetite regulation and weight regulation through the leptin-melanocortin pathway.

In this context, we have performed since 2022 a custom NGS targeted panel of 44 genes dedicated to monogenic and syndromic forms of severe and early onset obesity. The aim of this study is to assess the diagnostic yield of our approach in a diagnostic setting and to propose a clinical description of the whole cohort studied and a short description of individual cases to illustrate, secondarily, the perspectives and the need for guidance for genetic testing.

## Materials and methods

### Patients: inclusion criteria and data collection

Patients investigated through our targeted genetic obesity panel must present a severe early onset obesity starting ≤ 4 years of age as an isolated symptom or an obesity starting at 4 years or a few years later (in a pre-puberty stage) as a non-isolated symptom. Symptoms are assessed by the specialists who prescribe the analysis: (pediatric) endocrinologists but also other experts in the field of obesity management. Important, but not mandatory, criteria for genetic testing are the hyperphagia (defined by pathologic, insatiable hunger accompanied by abnormal food-seeking behaviors, including sometimes nocturnal eating) (8) or the lack of satiety. At that time, no mandatory dedicated and validated hyperphagia questionnaire was in use in Belgium, leaving this clinical criterion to the evaluation of the specialist. A family history is not mandatory, knowing that some genetic conditions are *de novo*.

A cohort of a total of 223 probands was tested between February 2022 and May 2024. Our cohort included only those patients for whom we received a well-filled clinical form that included specific clinical criteria and familial data (**Table 1**). For each patient, all the items have been recorded in an anonymized Excel database in accordance with the PGDR legacy and our internal hospital ethical legacy.

**Table 1:** Cohort description.

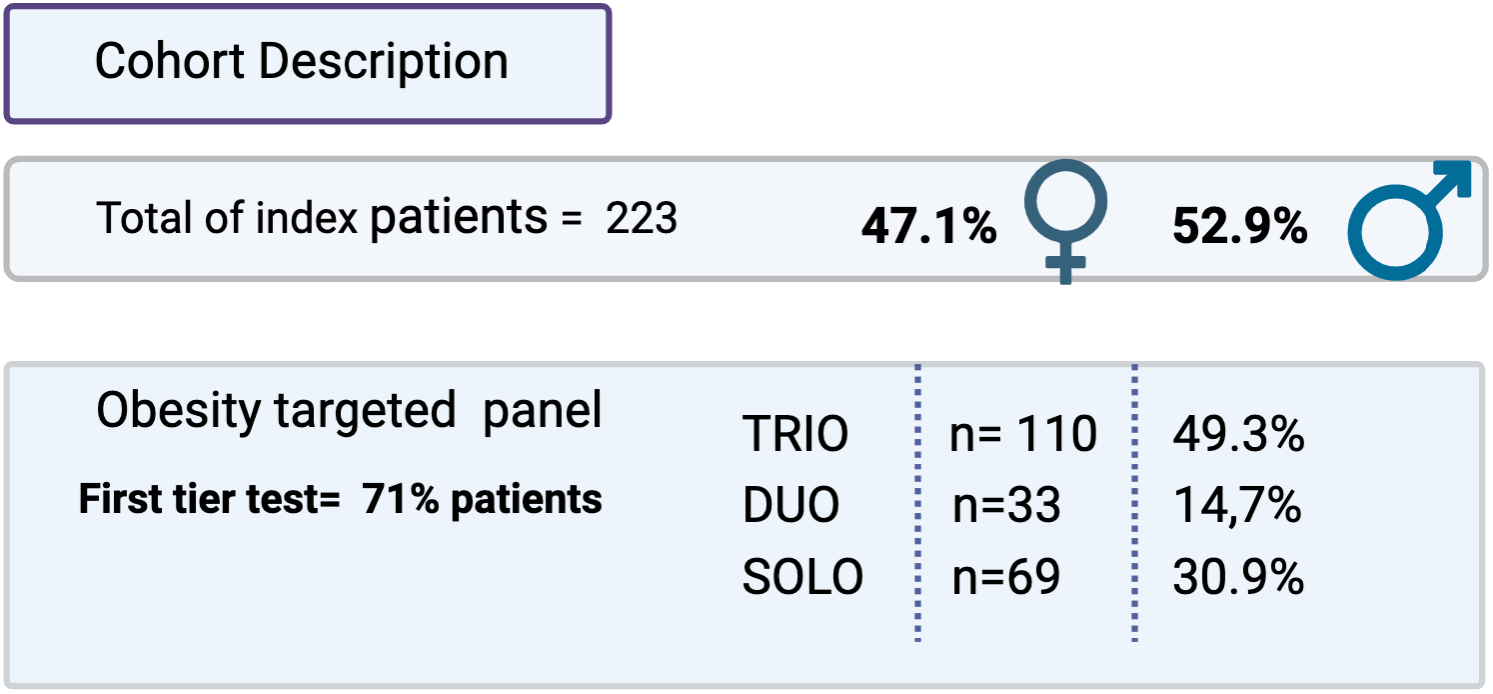

Number of index patients and genre repartition. Percentages of obesity targeted panels performed in trio, duo or solo.

Patients were included to perform solo, duo (proband and one parent), or trio (proband and both parents) analyses depending on the availability or not of the blood samples from the parents.

All the patients agreed to this study through the signatures of a consent form.

### Sequencing and bioinformatics methods

Our genetic test is an “in-house LDT test” developed from commercial kits. The targeted obesity gene panel contains 44 obesity-related genes: *ADCY3*, *ALMS1*, *BBS1*, *BBS2*, *BBS3 (ARL6)*, *BBS4*, *BBS5*, *BBS6 (MKKS)*, *BBS7*, *BBS8 (TTC8)*, *BBS9*, *BBS10*, *BBS11 (TRIM32)*, *BBS12*, *BBS13 (MKS1)*, *BBS14 (CEP290)*, *BBS15 (WDPCP)*, *BBS16 (SDCCAG8)*, *BBS17 (LZTFL1)*, *BBS18 (BBIP1)*, *BBS19 (IFT27)*, *BDNF*, *CREBBP*, *EP300*, *DYRK1B*, *GNAS*, *INPP5E*, *LEP*, *LEPR*, *MAGEL2*, *MC3R*, *MC4R*, *MRAP2*, *MYT1L*, *NTRK2*, *PCSK1*, *PHF6*, *POMC*, *RAB23*, *SETD2*, *SH2B1*, *SIM1*, *TBX3*, and *TUB*.

The list of the 44 genes was established based on an extensive literature review and a comparative review of the previously described worldwide obesity panel. Technically, each gene included in the panel had sufficient coverage by NGS sequencing. All the materials and methods used for the design of our custom panel were documented and accredited according to local regulations.

Genomic DNA was extracted from peripheral blood mononuclear cells in EDTA tubes using the Nucleomag Blood 200 µl kit (Macherey Nagel, Germany) on a MICROLAB STARlet (Hamilton, Reno, USA). DNA was quantified using NanodropOne (Thermo Fischer Scientific, MA, USA).

NGS sample preparation and enrichment was performed on 50 ng of DNA using the TWIST technology according to the manufacturer’s recommendations (TWIST Biosciences, CA, USA). The custom probes were designed to capture the exonic regions ± 14 bp of our targeted genes (captured region of about 134 kb). Samples were pooled by 16 before cluster generation and paired-end sequencing on a MiSeq using the MiSeq Reagent Kit V3 150 cycles (Illumina, San Diego, USA).

Raw data demultiplexing and generation of the FASTQ files were performed internally using “bcl2fastq” (Illumina). Sequencing reads were then analyzed via our internal bioinformatics pipeline (Humanomics, https://doi.org/10.5281/zenodo.13739359), which maps and prepares raw reads before inferring SNPs and INDELs following the GATK Best Practices. QC parameters are monitored following our internal diagnostics procedure and presented for interpretation using MultiQC.

### Variant interpretation

The analysis and interpretation of variants reaching a minimal 30X coverage were performed using Alissa Interpret Software 5.3 (Agilent Technologies, CA, USA) and according to ACMG interpretation variant guidelines (9) and their updates by the Sequence Variant Interpretation Working Group published on the ClinGen website.

## Results

Our total cohort includes 223 patients. (**Table 1**) For 110 patients, we had trio samples, and for 33 patients, we collected duo samples. Thirty percent of samples came from national external centers, and 70% came from the geographical area linked to our university location.

The clinical dataset from all the cohorts of the 223 recruited patients reveals that 45% (*N* = 102/223) are described as hyperphagic. Regarding the other patients (121 of 223), the eating behavior was not clearly mentioned to allow an appropriate interpretation.

To evaluate epidemiologically the type of early onset obesity tested, patients were divided into three groups: weight gain started ≤ 1 year of age, between 1 and 4 years of age, or after 4 years of age. Data are missing for seven of the 223 patients. The first group represents 23% of the cohort, the second 47%, and the third 30%. (**Table 2**) Associated symptoms or features are detailed in **Table 2**. Specifically, among the 2.3% of patients reported with red hair, no *POMC* variants have been detected. Only two patients presented with retinitis pigmentosa. One of them has a confirmed diagnosis of BBS. The second had all the criteria for BBS without molecular confirmation at that time. Overgrowth was reported for 10% of the cohort (22 of 220), and no positive cases were found among these 10%. A subset of 3.7% of this last group of patients was found to be investigated also by an overgrowth-targeted panel. More generally, 36% of the cohort was reported with neurological concerns, including motor delay (11%), language delay (19%), and intellectual disabilities (16%). Neurological features were found in the same ratio between the patients with positive or negative results. Additional genetic investigations for neurodevelopmental troubles have been performed using an array CGH in 37% (79 of 214), an intellectual disability panel in 7.4% (16 of 216), and a dedicated test for *FMR1* in 12.5% (**Table 3**).

**Table 2:** Auxological and clinical descriptive features of the total cohort.

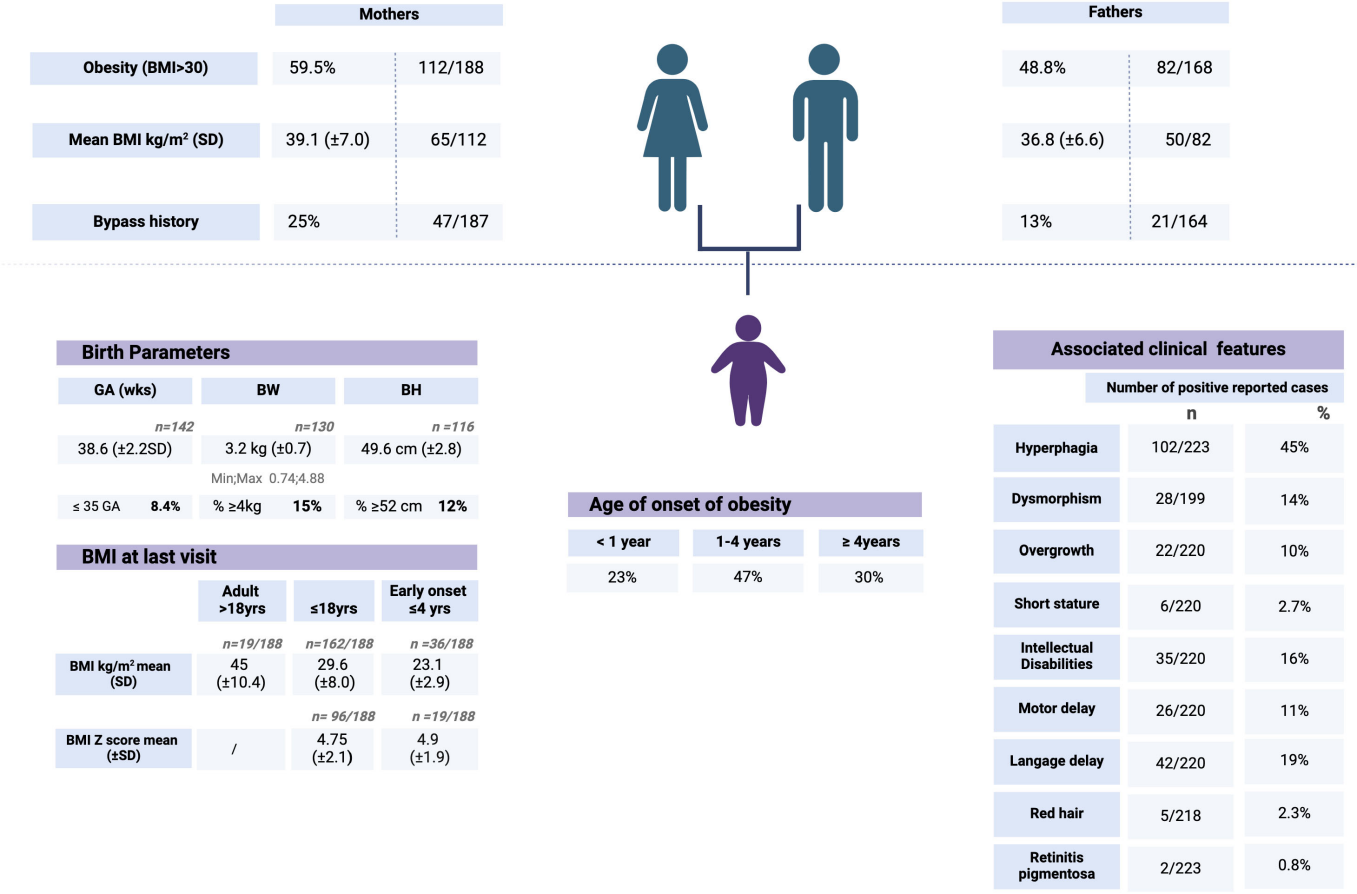

Auxological data. For each patient, age of onset of obesity, birth parameters, BMI at last visit and associates’ symptoms were collected.

To assess the timing of early onset obesity, patients were divided into three groups: weight gain started ≤1 year of age (23%), between 1 and 4 years of age (47%), or after 4 years of age (30%).

Birth parameters show that 8.4% of the patients were born prematurely (<35GA). To estimate the number of patients in the criteria of macrosomia/overgrowth at birth, we calculated that 15% presented a BW >4kg and 12% a BH >52cm.

For each patient, available data from the parents included BMI >30, exact BMI at the time of sample collection and bypass history. Each item was calculated in percentage or mean BMI for both groups of mothers and fathers.

Clinical descriptive features. For each patient, associated symptoms were described by the prescribers and/or reviewed by our team. The list of features is described with the number (*n*) and the percentage (%) of positive patients for each item and for each group (total cohort, patients with a positive genetic result and patients with a negative genetic result).

**Table 3:** Number and type of additional genetic investigations performed in the cohort.

Number and type of additional genetic investigations		
	%	n
Array CGH	37%	79/214
ID gene panel	7.4%	16/216
Overgrowth gene panel	3.7%	8/215
FMR1 gene analysis	12.5%	27/216
Prader-Willi Syndrome	9.1%	19/208
Whole Exome Sequencing	2.8%	6/212
Angelman		3
BBS targeted gene panel		3
BWS		3
Temple syndrome		6
MODY panel		3
Metabolic work-up		4

Parents themselves are reported with the general criteria of being affected by obesity (BMI > 30 with no precision) in 59.5% (112 of 188) of the mothers and 48.8% (82 of 168) of the fathers. The exact BMI (normal or in the criteria of obesity) was specifically recorded for 82 fathers and 92 mothers, of whom 51 and 65, respectively, had a BMI ≧ 30 kg/m^2^. The median BMI for the fathers affected by obesity is 35.3 kg/m^2^ (*M* = 36.8; *SD* = 6.64), and the median BMI for the mothers affected by obesity is 38.05 kg/m^2^ (*M* = 39.1; *SD* = 7.03). Only one father has been reported to have died (unexplained cause).

As an indicator of familial severe obesity, parental history of bypass surgery was reported in 13% (*n* = 21/164) of fathers and 25% of mothers (*n* = 47/187).

The diagnostic yield of our targeted panel is 3.1% (7 of 223) (**Table 4A**). The seven positive results identified in six families are listed in **Table 4B**. Our panel was performed as a first-tier analysis in 71.5% of the cases (156 of 218). The genetic test for Prader-Willi was additionally requested for 9.1% (19 of 218) of the patients and showed no positive results.

**Table 4a:** Results summary showing the number of variants (P/LP/VUS) identified in the cohort through the different genetic tests.

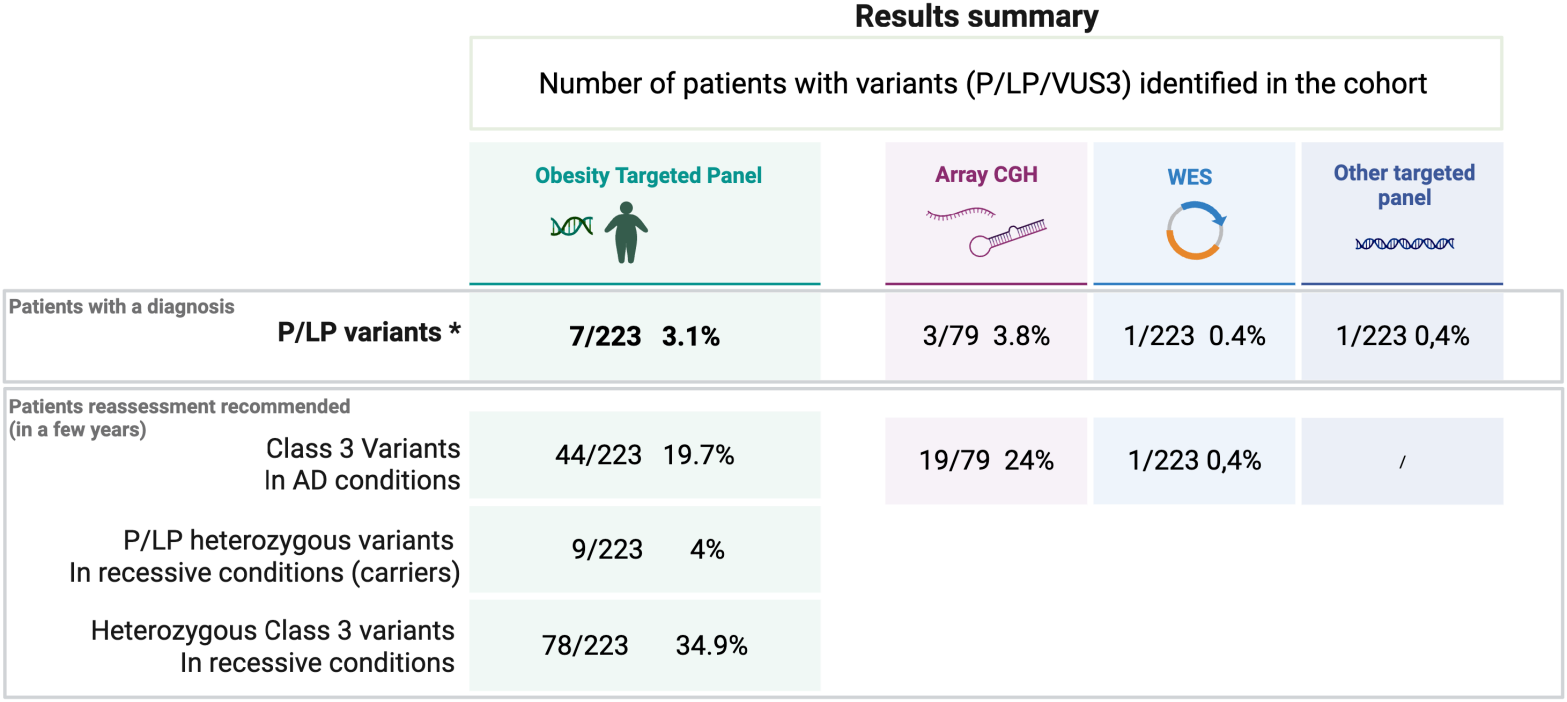

The diagnosis yield for the obesity panel is 3.1%. The total diagnosis yields all tests included for the whole cohort is 4.9.%. The results discriminate furthermore the number of patients who are carriers of a heterozygous LP/P in a gene associated with a recessive condition and the number of patients for whom the genetic test found at least one VUS in respectively autosomal conditions and recessive conditions.

*The P/PL variants reported for the patients with a confirmed diagnosis are in heterozygous state for autosomal conditions or in a homozygous or composite heterozygous state for recessive conditions.

**Table 4b:** Molecular results identified for each patient (total of 11 positive case) and listed according to the genetic test performed.

Patient ID	Results from the targeted panel	
1	Class 4 variant c.68G>C, p.(Arg23Pro) homozygous in the *BBS2* gene	NM_031885.4 (BBS2)
	And a classs 3 variant c.884G>A, p.(Arg295Gln) in the *BBS4* gene	NM_033028.5 (BBS4)
2	Class 4 variant c.535delG, p.(Val179Phefs*39) in the *MC4R* gene (NM_005912.3) at a heterozygous status. Maternally inherited.	NM_005912.3
3	Class 4 variant c.181G>T, p.Glu61* in the MC4R gene at a heterozygous status. Parental analyses not performed.	NM_005912.3 NM
4	Class 4 variant c.240C>A, p.(Tyr80*) in the MC4R gene at a heterozygous status	NM_005912.3
5	Class 4 variant c.91_92delinsTA, p.(Gly31*) in the *MRAP2* gene at a heterozygous status. Maternally inherited.	NM_138409.4
6	Class 4 variant c.91_92delinsTA, p.(Gly31*) in the *MRAP2* gene at a heterozygous status. Maternally inherited.	NM_138409.4
7	Deletion of the exon 1 of the *SEZ6L2* gene at a heterozygous status (region of the deletion 16p11.2)	NM_012410
Array CGh results		
7	arr[GRCh37] 7q36.3(158269643_158599209)x3 pat,16p11.2(29592783_30190568)x1 pat	
8	arr[GRCh37] 16p12.2(21837492_22407931)x1 dn	
9	arr[GRCh37] 9p21.1(28219132_28377937)x1 including *LINGO2*.	
ID panel		
10	Class 4 variant c.[1021T>G];[=],p.[Cys341Gly];[=] in the DDX3X gene at a heterozygous status. De novo.	NM_001193416.3
WES		
11	Class 5 variant c.1207_1216delinsCACTGTGACA; p. Lys406ThrfsTer3 homozygous in the *P4HTM* gene.	NM_177939.3

However, additional positive results were found in the cohort: array CGH revealed positive results for three patients (**Table 4A**), but we know that one of these three patients was initially identified with a 16p11.2 deletion by the obesity-targeted panel. For this specific case, array CGH was completed to investigate additional chromosomal abnormalities.

A pathologic variant in *DDX3X* was found through an intellectual disabilities WES panel. Finally, six patients from the cohort were investigated by a WES, with one positive result identified for one of the 6 patients: a young girl with HIDEA Syndrome previously published in 2023 (10).

Out of our cohort, seven patients presented an LPV or PV in the genes *MC4R*, *MRAP2*, and *BBS2*, and a 16p11.2 deletion for one patient. (**Table 4B**) The median BMI for these positive cases is 35.2 kg/m^2^ (*SD* = 10.4, *n* = 6/7), and the median age at the time of genetic investigations is 11.66 years (*SD* = 6.6). Five out of the seven positive patients presented an early onset of obesity starting ≤ 4 years; more specifically, two of them started ≤ 1 year.

A carrier status was found for nine patients (9/223 = 4%) (**Table 4A**). They present either heterozygous LPV or PV in genes related to recessive OMIM conditions. The genes encountered in this subgroup are *LZTFL1*, *BBS7*, *BBS1*, *CEP290*, *POMC*, *LEP*, and *PCSK1*.

Regarding the percentage of VUS found in our gene panel, 44 patients (19.7%) present at least one VUS in genes associated with conditions with an autosomal dominant inheritance, and 78 patients (34.9%) of the cohort present at least one VUS associated with a recessive condition. (**Table 4A**) Lack of segregation analyses or lack of literature consensus are the main reasons why the pathogenicity of these variants remains uncertain until now. Reevaluation of these 122 (54.7%) patients [considering patients with a VUS for both AD (19.7%) and AR (34.9%) conditions] might be proposed in the next few years to perform a reevaluation of the genotype-phenotype correlations and expand the genetic testing thanks to the new genomic technologies that should be available in a diagnostic setting.

Regarding specifically the BBS variants, 38 patients (17%) encountered at least one BBS VUS, and seven LPV/PV were found in seven additional patients. These seven patients are heterozygous, and no second variant in BBS genes was identified. For three patients, the clinical suspicion remains highly significant and investigations encompassing intronic region analyses are still ongoing to try to identify a second variant for one patient. Segregation analyses are still lacking for two patients.

## Discussion

The diagnosis of monogenic obesity in children remains a current challenge. In that view, since 2022, a targeted panel of 44 genes specifically dedicated to the genetic forms of severe and early onset obesity has been implemented in the CHU of Liège, Belgium. 223 index probands were tested. Seven patients were found with LPV or PV, which represents a diagnostic yield of 3.1%. Nevertheless, this descriptive series also highlights a significant number of heterozygous class 3 variants (19.7% related to autosomal dominant conditions and 46.6% to recessive conditions) and 4% of heterozygous carriers of an LPV or PV in a gene encountered in recessive conditions.

**Figure 1:**
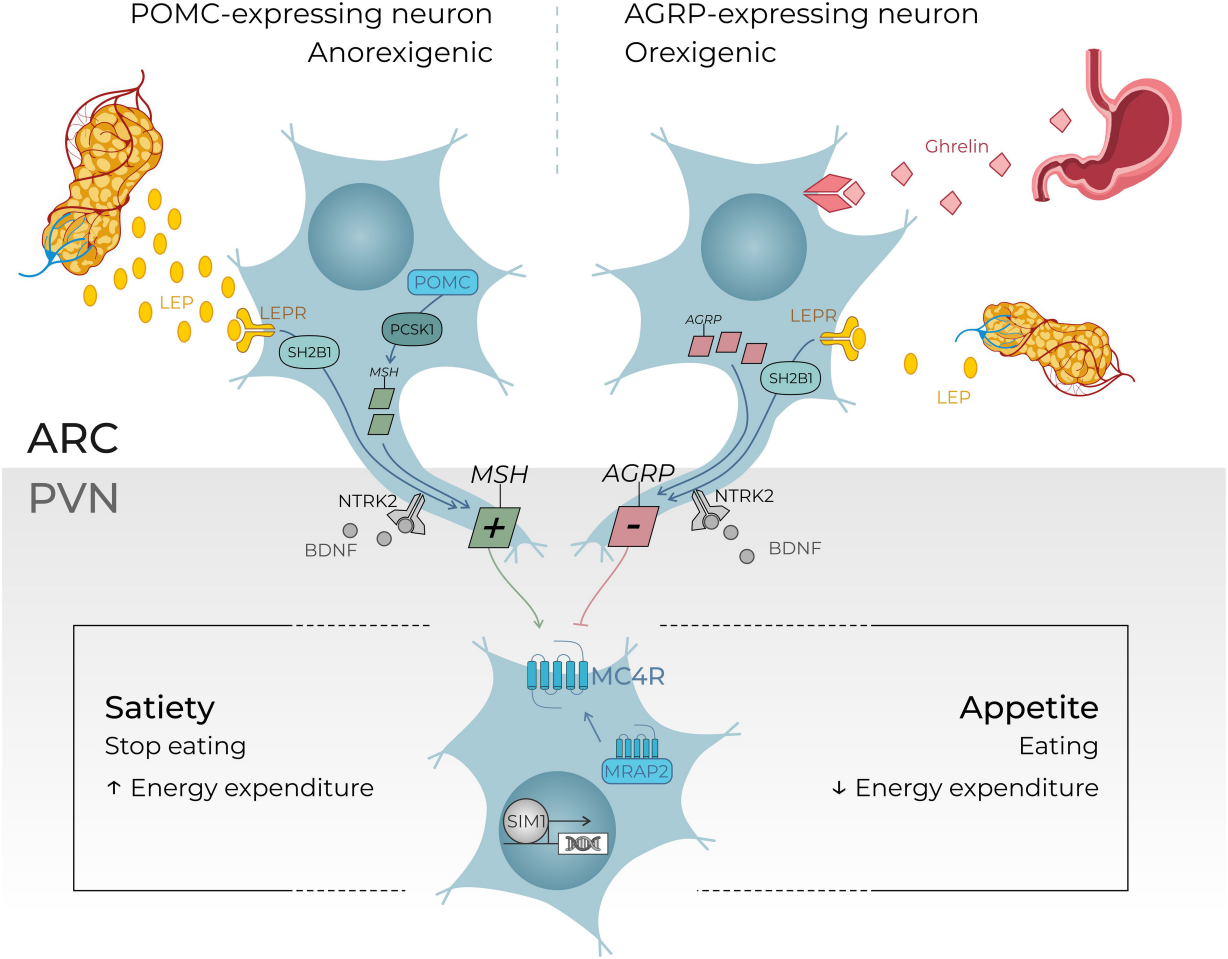
An overview of the leptin–melanocortin pathway in the hypothalamus. Leptin is secreted in the white adipose tissue. Leptin levels depend on the “fed status”: leptin levels increase in case of refeeding after food starvation and leptin levels decrease in case of food deprivation. Leptin hormone peptides act on the hypothalamus where POMC-expressing neurons and agouti-related protein (AGRP)–expressing neurons are located, more precisely in the arcuate nucleus. These neurons send a signal to the MC4R-expressing neurons in the paraventricular nucleus of the hypothalamus (PVN) which controls through their central neural projections *in fine* the level of appetite or satiety. BDNF (brain-derived neurotrophic factor) is thought to be an actor in this pathway, through its binding to NTRK2 (neurotrophic receptor tyrosine kinase 2) leading to a regulation in the synaptic plasticity of neurons, including those present in the ARC and PVN. The transcription factor SIM1 is also essential for the correct development of the PVN. This overview gives comprehensive examples of regulator genes investigating through our targeted gene panel. (5, 11–15). +, agonist; −, antagonist; LEPR, leptin receptor; MRAP2, melanocortin receptor accessory protein 2; MSH, melanocyte-stimulating hormone; SH2B1, SH2B adaptor protein 1. ARC, arcuate nucleus; AgRP, agouti related peptide; NPY, neuropeptide Y; POMC, propiomelanocortin; αMSH, α-melanocyte stimulating hormone; MCR, melanin-concentrating hormone receptor; SST, somatostatin.

**Figure 2:**
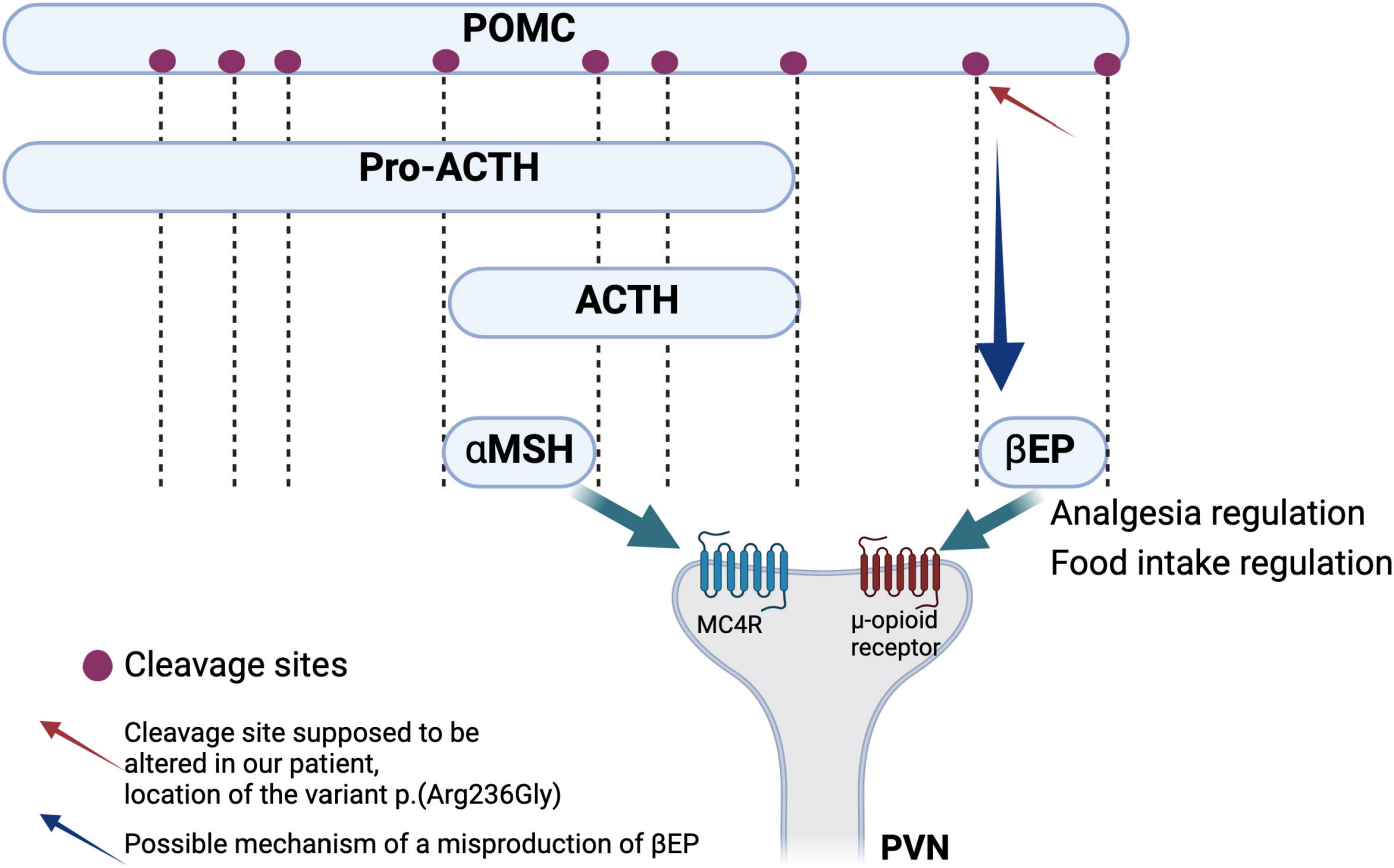
Simplified schema of the processing of POMC leading to the generation of functional peptides including αMSH and ß-endorphins. These two peptides act through the binding to two different types of receptors, respectively MC4R and μ-opioid receptor. Red arrow shows the cleavage site that is thought to be altered in our patient hypothesizing an alteration of the leptin melanocortin pathway through the alteration of the ß-endorphins action which should probably lead to food intake dysregulation but also to analgesia and reward process dysregulation (17–19).

The seven positive cases diagnosed in our cohort present variants in *MC4R*, *MRAP2*, and *BBS* genes and a 16p11.2 deletion. All these molecular alterations lead to a dysregulation of the appetite control, mainly through a disturbance of the hypothalamic leptin-melanocortin pathway, the main cause of monogenic non-syndromic obesities (**Figure 1**). Clinically, it is well known that a deficiency of *POMC* (bi-allelic variants) leads to hyperphagia, lower resting metabolic rate, and severe obesity with cutaneous pigmentation abnormalities (red hair and pale skin) (16). However, the impact of heterozygous *POMC* variants on obesity is still unclear. A recent publication of 2023– (17) concludes that heterozygous pathogenic *POMC* variants do not contribute to monogenic obesity but that they slightly increase the BMI (17). Further data will probably be more accurate in the next few years considering the subsequent question of the relevance (or not) of using new treatments such as setmelanotide in this indication. In our cohort, we found three patients with *POMC* VUS for whom the interpretation should improve with better segregation data, but familial DNA samples are not available. One of them presents, nevertheless, a highly questioning VUS that raises the question of the impact of specific variants located on cleavage sites of the POMC protein. (**Figure 2**) POMC is cleaved by pro-hormone convertases at dibasic sites, which are generally well conserved between species (18). The expression of the *POMC* gene is based on complex mechanisms that regulate the release of POMC-derived peptides such as MSH, ACTH, and ß-endorphins (**Figure 2**). Our patient presents the variant *POMC* c.706C>G, p.(Arg236Gly), which is located on the cleavage site involved in the generation of ß-endorphins. ß-endorphins are known to play a role in the regulation of analgesia but also in the regulation of food intake through their specific activation of the µ-opioid receptors and not the MC4R. A study of mice with deletion of ß-endorphins reveals that male mice were obese and hyperphagic (19). In addition to this anorexigenic role, ß-endorphins are also involved in the positive regulation of the appetite through the reward behavior system. Processing of POMC is therefore a complex and subtle pathway that might need more detailed knowledge to better appreciate the functional consequences of each specific variant. Notably, in the case of a variant leading to a specific ß-endorphin deficiency, a treatment such as MC4R-agonist would not be indicated.

**Figure 3:**
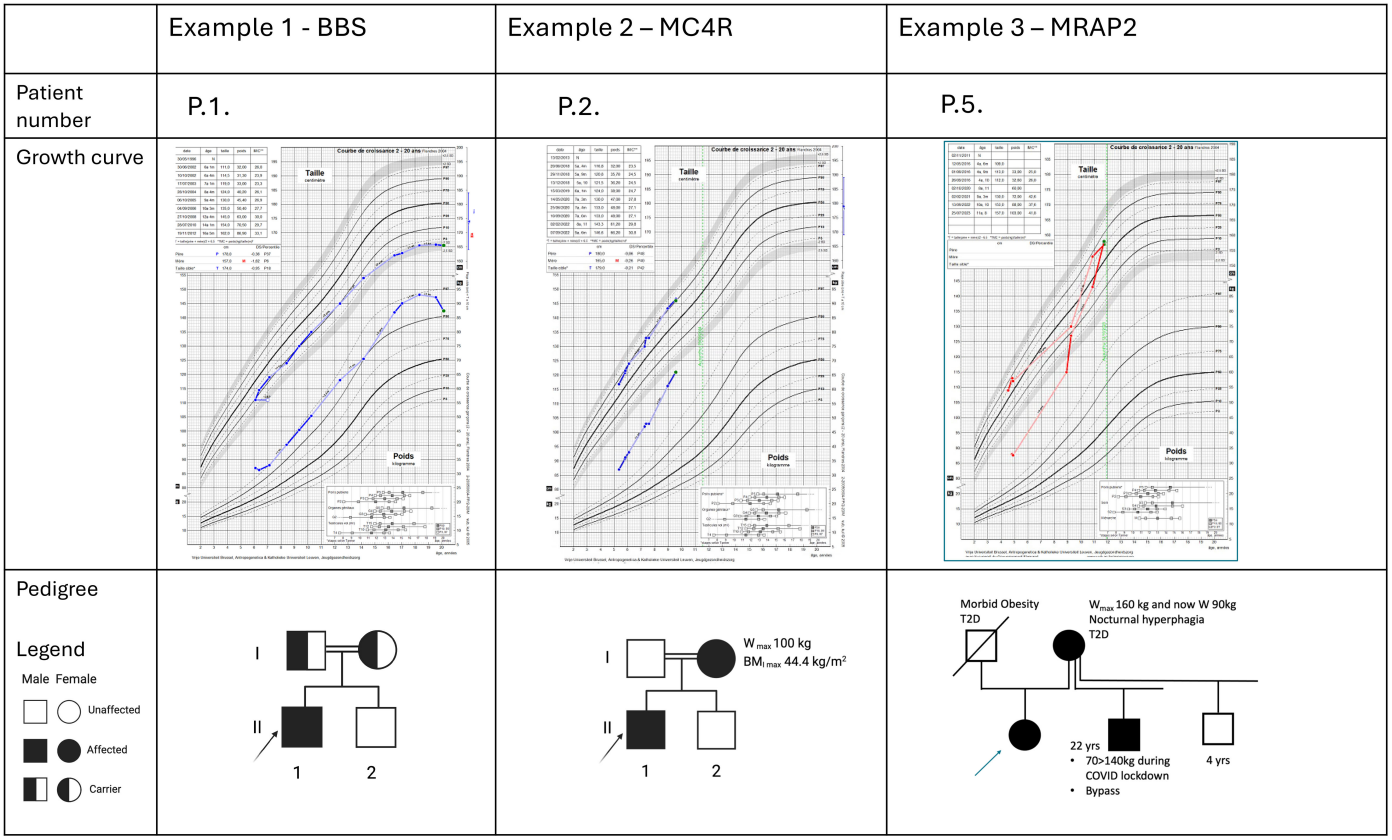
Description of three illustrative positive cases. For each patient, the familial pedigree shows the inheritance pattern. The growth curves reveal for the three cases a severe obesity with early onset and a weight relatively stabilized at adult age for the case P1. At last evaluation, they have all a significant pathologic BMI. P.1 presents associated features typically in accordance with a BBS syndrome. The two other cases present no discriminative clinical pattern that may potentially orient the diagnosis hypotheses.

Interestingly*, MC4R* remains the first cause of monogenic obesity, with an estimated prevalence of 5% and 2% in the obese pediatric and adult cohorts, respectively (16). MC4R is expressed in the hypothalamus, brain, muscle, adipocytes, and astrocytes and is involved not only in energy homeostasis and food intake but also in anti-inflammatory regulation, drug tolerance, and sexual behavior (20, 21). Patients with homozygous variants are extremely rare in Europe; a few consanguineous families are described showing a highly severe phenotype with a very early onset of hyperphagia. In contrast, heterozygous patients present a wide phenotypical spectrum ranging from very early onset hyperphagia to minor excess weight in adulthood. Three patients with an LPV or PV in *MC4R* were detected in our cohort (3/223 = 1.35%): two *MC4R* pathogenic variants previously published (22, 23) and one *MC4R* LP variant c.535delG (p.(Val179Phefs*39) never reported until now. (Patient 2, **Figure 3**). Our diagnosis yield is probably lowered because, in case of suspicion of MC4R, Belgian clinicians have the possibility to prescribe *MC4R*-targeted tests, leading to a statistical estimation of the incidence of MC4R-positive patients that is difficult without a specific registry.

Another key component in the leptin-melanocortin pathway is the melanocortin 2 receptor accessory protein 2 (MRAP2). (**Figure 1**) Since 2023, MRAP2 is known to be required for the localization of MC4R to the primary cilia and the function of MC4R neurons (24), an emerging knowledge providing new insights in recent theories linking energy homeostasis and primary cilia. In this perspective, research for new candidate genes should also be oriented to genes controlling the localization of MC4R to the primary cilia (24). Loss-of-function pathogenic variants in *MRAP2* are related to monogenic hyperphagic obesity associated with hyperglycemia and hypertension, contrasting with the other monogenic forms of obesity that present generally with low blood pressure and normal glucose tolerance. As deficiency in MRAP2 partly affects the MC4R pathway, the subsequent energy homeostasis dysregulation and obesity in *MRAP2*-deficient subjects might be theoretically improved by an MC4R-agonist treatment (25). However, further studies are needed to better delineate mechanisms and efficacy of this therapeutic option in *MRAP2*-related obese patients. In our cohort, a new MRAP2 variant was found in two related probands: *MRAP2* loss-of-function LPV c.91_92delinsTA, p.(Gly31*) (**Figure 3**).

Early onset obesity causes are evidently broader, encompassing environmental, hormonal, and oligogenic factors. Among these oligogenic predispositions, the MC4R pathway seems to play a key role, and the future would be to consider both genetic studies, monogenic and oligogenic, of our patients in diagnostic settings. For now, current estimations from different studies dedicated to monogenic etiologies suggest that 5% of the patients with severe early onset obesity are linked to a monogenic condition related to the melanocortin pathway. From one point of view, a mean of 5% for the efficiency rate for the targeted panels may be discussed as underestimated due to limited access to genetic investigations for a wide range of patients. Medical compliance and socioeconomic status should be cited as two factors of under evaluation for this category of patients. On the other hand, the 5% diagnostic yield should also be interpreted with caution, as it might be overestimated. A thorough analysis of previously published series reveals an inflated rate of positive results due to inconsistencies between studies and differing criteria for variant classification, which could lead to the misclassification of variants of uncertain significance (VUS) as positive results (26–28). In our cohort, the diagnostic yield for the targeted panel itself was 3.1%. However, a total of 4.9% of diagnoses in our cohort were confirmed through additional genetic investigations performed on highly suspicious cases. (**Table 4B**) Moreover, our results found that 54.7% of the patients had at least one heterozygous VUS; specifically, 19.7% of VUS were related to autosomal conditions and 46.6% to recessive conditions. For a limited number of the patients of the cohort, stronger genotype-phenotype correlations or additional genetic investigations in a research setting are still ongoing due to a high suspicion of variant pathogenicity. Nevertheless, a systematic re-evaluation might be recommended for all the patients presenting at least one VUS (54.7%) in a few years to expand the potential for new positive results. Notably, we hypothesize that the future availability in a diagnostic setting of genomic testing technologies (WGS) or long-read sequencing should be key next steps to improve the detection of second variants in bi-allelic conditions.

In the field of monogenic obesity, depending on laboratory resources, a targeted panel provides advantages of relatively short turnaround times, avoidance of incidental findings, and higher sequencing quality (by improving the coverage) (29). For these reasons, investigations through a targeted panel as a first-tier analysis remain currently considered to be a more effective screening method than WES in the population of patients with severe early onset obesity.

In pediatrics, it is currently well established that obesity starting ≤ 4 years must be investigated with a genetic test (30, 31). Our diagnosis rate calculated for our cohort subgroup of obesity starting ≤ 4 years is 3.3%, which is not significantly discriminant compared to obesity starting > 4 years. This cutoff age of 4 years for the onset of obesity might not be systematically used as a stringent criterion in clinical practice for requesting genetic testing. Among our seven positive cases, three are presented (**Figure 3**) to illustrate the types of growth curves observed in childhood genetic obesity and the challenges in establishing a cutoff based on clinical data and growth parameters for genetic testing. Furthermore, our findings do not reveal any distinguishing clinical features between the seven positive cases and the rest of the cohort (**Table 2**). Similarly, the statistical comparisons between the group of patients presenting a VUS and the rest of the cohort did not reveal any significant differences. Larger sample sizes should be recommended to enhance the statistical performance test. Nonetheless, the variability and minimal clinical differences among monogenic disorders reinforce the notion that clinical criteria alone are insufficient to restrict access to genetic testing.

Regarding the young adult patients, no consensual recommendations have yet been published on this topic. From our perspective, genetic investigations should be implemented more widely for all the young adults (especially between 18 and 25 years) with an extremely severe BMI (BMI ≥ 40 kg/m^2^) and a medical history of prepubertal childhood obesity with no evident explanation (no adverse drug effect, no diet imbalance).

More than an added value, the confirmation of monogenic obesity should imply therapeutic perspectives. For example, the bariatric surgery effectiveness in patients with monogenic obesity remains debated. Genetic alterations, such as MC4R dysfunction, disrupt appetite regulation, potentially explaining the observation of differences in the long-term outcome in percentage of weight loss in comparison to patients with a non-genetic obesity (28). The new genetics knowledge has also opened the field for current and future therapies, as seen initially with the treatment of congenital leptin deficiency by recombinant leptin (32). *POMC*, *PCSK1*, and *LEPR* deficiencies and Bardet-Biedl syndrome (*BBS*) can be counter-regulated by the MC4R agonist setmelanotide (33, 34). One remaining question is to determine the impact of MC4R agonists in patients with heterozygous *POMC* variants, identifying a targeted group of responders based on variant types. These variants affect protein cleavage pathways (ß-endorphins or α-MSH), paving the way for more specific targeted therapy research.

However, improving genetic diagnosis for early onset obesity is the way to offer appropriate, preventive, and dedicated care management according to the genetic etiology and its associated risks (e.g., ophthalmopathy, renal failure, metabolic disturbances…).

## Conclusion

Monogenic causes of early onset obesity are still challenging to diagnose due to a lack of clinical discriminant criteria. Current guidance for clinicians proposes to identify candidates for genetic investigations among those patients with early onset obesity and hyperphagia. In our experience, based on that guidance, the diagnostic yield for a genetic diagnosis is 3.1% for the total cohort, increasing to 4.9% with additional molecular investigations. However, 19.7% and 46.6% of variants, respectively, associated with autosomal or recessive conditions, remain of unknown significance, highlighting the need to reevaluate systematically our patients in a few years in a diagnostic setting and to offer further research testing in selected cases. Our literature review underlines the discrepancies between the previous reported series and the non-uniformization for the reporting of the positive results. In the era of precision medicine, strengthening expertise in genetic obesity is essential for accurate diagnoses and to orient our patients through effective targeted (future) therapies.

## Data Availability

The original contributions presented in the study are included in the article/supplementary material. Further inquiries can be directed to the corresponding author.

## References

[1] Drieskens S Charafeddine R Gisle L. Enquête de santé 2018: Etat nutritionnel. Bruxelles, Belgique: Sciensano; Report number: D/2019/14.440/62 . Available online at: www.enquetesante.be (Accessed June 01, 2025).

[2] Skinner AC Ravanbakht SN Skelton JA Perrin EM Armstrong SC. Prevalence of obesity and severe obesity in US children, 1999-2016. Pediatrics. (2018) 141:e20173459. doi: 10.1542/peds.2017-3459, PMID: 29483202 PMC6109602

[3] Chung YL Rhie YJ. Severe obesity in children and adolescents: metabolic effects, assessment, and treatment. J Obes Metab Syndr. (2021) 30:326–35. doi: 10.7570/jomes21063, PMID: 34924365 PMC8735819

[4] Marenne G Hendricks AE Perdikari A Bounds R Payne F Keogh JM. Exome sequencing identifies genes and gene sets contributing to severe childhood obesity, linking PHIP variants to repressed POMC transcription. Cell Metab. (2020) 31:1107–1119.e12. doi: 10.1016/j.cmet.2020.05.007, PMID: 32492392 PMC7267775

[5] Farooqi IS. Monogenic human obesity syndromes. In: Handbook of clinical neurology, vol. 181. Amsterdam, Netherlands: Elsevier (2021). p. 301–10. doi: 10.1016/B978-0-12-820683-6.00022-1, PMID: 34238466

[6] Dubern B Mosbah H Pigeyre M Clément K Poitou C. Rare genetic causes of obesity: Diagnosis and management in clinical care. Ann Endocrinol. (2022) 83:63–72. doi: 10.1016/j.ando.2021.12.003, PMID: 34953778

[7] Forsythe E Beales PL. Bardet–biedl syndrome. Eur J Hum Genet. (2013) 21:8–13. doi: 10.1038/ejhg.2012.115, PMID: 22713813 PMC3522196

[8] Heymsfield SB Clément K Dubern B Goldstone AP Haqq AM Kühnen P. Defining hyperphagia for improved diagnosis and management of MC4R pathway–associated disease: A roundtable summary. Curr Obes Rep. (2025) 14:13. doi: 10.1007/s13679-024-00601-z, PMID: 39856371 PMC11762201

[9] Richards S Aziz N Bale S Bick D Das S Gastier-Foster J. Standards and guidelines for the interpretation of sequence variants: a joint consensus recommendation of the American College of Medical Genetics and Genomics and the Association for Molecular Pathology. Genet Med Off J Am Coll Med Genet. (2015) 17:405–24. doi: 10.1038/gim.2015.30, PMID: 25741868 PMC4544753

[10] Harvengt J Lumaka A Fasquelle C Caberg JH Mastouri M Janssen A. HIDEA syndrome: A new case report highlighting similarities with ROHHAD syndrome. Front Genet. (2023) 14:1137767. doi: 10.3389/fgene.2023.1137767, PMID: 37035730 PMC10073441

[11] Rohde K Keller M Poulsen L la C Blüher M Kovacs P Böttcher Y. Genetics and epigenetics in obesity. Metab - Clin Exp. (2019) 92:37–50. doi: 10.1016/j.metabol.2018.10.007, PMID: 30399374

[12] Da Fonseca ACP Mastronardi C Johar A Arcos-Burgos M Paz-Filho G. Genetics of non-syndromic childhood obesity and the use of high-throughput DNA sequencing technologies. J Diabetes Complications. (2017) 31:1549–61. doi: 10.1016/j.jdiacomp.2017.04.026, PMID: 28735903

[13] Loos RJF Yeo GSH. The genetics of obesity: from discovery to biology. Nat Rev Genet. (2022) 23:120–33. doi: 10.1038/s41576-021-00414-z, PMID: 34556834 PMC8459824

[14] Baldini G Phelan KD. The melanocortin pathway and control of appetite-progress and therapeutic implications. J Endocrinol. (2019) 241:R1–R33. doi: 10.1530/JOE-18-0596, PMID: 30812013 PMC6500576

[15] Kumar U Singh S. Role of somatostatin in the regulation of central and peripheral factors of satiety and obesity. Int J Mol Sci. (2020) 21:2568. doi: 10.3390/ijms21072568, PMID: 32272767 PMC7177963

[16] Mahmoud R Kimonis V Butler MG. Genetics of obesity in humans: A clinical review. Int J Mol Sci. (2022) 23:11005. doi: 10.3390/ijms231911005, PMID: 36232301 PMC9569701

[17] Le Collen L Delemer B Poitou C Vaxillaire M Toussaint B Dechaume A. Heterozygous pathogenic variants in POMC are not responsible for monogenic obesity: Implication for MC4R agonist use. Genet Med Off J Am Coll Med Genet. (2023) 25:100857. doi: 10.1016/j.gim.2023.100857, PMID: 37092539

[18] Harno E Gali Ramamoorthy T Coll AP White A. POMC: the physiological power of hormone processing. Physiol Rev. (2018) 98:2381–430. doi: 10.1152/physrev.00024.2017, PMID: 30156493 PMC6170974

[19] Appleyard SM Hayward M Young JI Butler AA Cone RD Rubinstein M. A role for the endogenous opioid β-endorphin in energy homeostasis. Endocrinology. (2003) 144:1753–60. doi: 10.1210/en.2002-221096, PMID: 12697680

[20] Singh RK Kumar P Mahalingam K. Molecular genetics of human obesity: A comprehensive review. C R Biol. (2017) 340:87–108. doi: 10.1016/j.crvi.2016.11.007, PMID: 28089486

[21] Semple E Hill JW. Sim1 neurons are sufficient for MC4R-mediated sexual function in male mice. Endocrinology. (2017) 159:439–49. doi: 10.1210/en.2017-00488, PMID: 29059347 PMC5761591

[22] Lubrano-Berthelier C Dubern B Lacorte JM Picard F Shapiro A Zhang S. Melanocortin 4 receptor mutations in a large cohort of severely obese adults: prevalence, functional classification, genotype-phenotype relationship, and lack of association with binge eating. J Clin Endocrinol Metab. (2006) 91:1811–8. doi: 10.1210/jc.2005-1411, PMID: 16507637

[23] Brumm H Mühlhaus J Bolze F Scherag S Hinney A Hebebrand J. Rescue of melanocortin 4 receptor (MC4R) nonsense mutations by aminoglycoside-mediated read-through. Obes Silver Spring Md. (2012) 20:1074–81. doi: 10.1038/oby.2011.202, PMID: 21738238

[24] Bernard A Naharros IO Bourgain-Guglielmetti F Ciprin J Yue X Zhang S. The single pass membrane protein MRAP2 regulates energy homeostasis by promoting primary cilia localization of the G protein-coupled receptor MC4R. Neuroscience. (2020) 523:113–25. doi: 10.1101/2020.11.13.382325

[25] Baron M Maillet J Huyvaert M Dechaume A Vatin V Boutry R. Loss-of-function mutations in MRAP2 are pathogenic in hyperphagic obesity with hyperglycemia and hypertension. Nat Med. (2019) 25:1733–8. doi: 10.1038/s41591-019-0622-0, PMID: 31700171 PMC6858878

[26] Kleinendorst L Massink MPG Cooiman MI Savas M van der Baan-Slootweg OH Roelants RJ. Genetic obesity: next-generation sequencing results of 1230 patients with obesity. J Med Genet. (2018) 55:578–86. doi: 10.1136/jmedgenet-2018-105315, PMID: 29970488

[27] Mohammed I Haris B Al-Barazenji T Vasudeva D Tomei S Al Azwani I. Understanding the genetics of early-onset obesity in a cohort of children from Qatar. J Clin Endocrinol Metab. (2023) 108:3201–13. doi: 10.1210/clinem/dgad366, PMID: 37329217 PMC10655519

[28] Bonetti G Dhuli K Ceccarini MR Kaftalli J Samaja M Precone V. Next-generation sequencing of a large gene panel for outcome prediction of bariatric surgery in patients with severe obesity. J Clin Med. (2022) 11:7531. doi: 10.3390/jcm11247531, PMID: 36556146 PMC9783894

[29] Yu H Yu H Zhang R Zhang Y Wang Y Li J. Targeted gene panel provides advantages over whole-exome sequencing for diagnosing obesity and diabetes mellitus. J Mol Cell Biol. (2023) 15:mjad040. doi: 10.1093/jmcb/mjad040, PMID: 37327085 PMC10847719

[30] Styne DM Arslanian SA Connor EL Farooqi IS Murad MH Silverstein JH. Pediatric obesity-assessment, treatment, and prevention: an endocrine society clinical practice guideline. J Clin Endocrinol Metab. (2017) 102:709–57. doi: 10.1210/jc.2016-2573, PMID: 28359099 PMC6283429

[31] Cuda SE Censani M. Assessment, differential diagnosis, and initial clinical evaluation of the pediatric patient with obesity: An Obesity Medical Association (OMA) Clinical Practice Statement 2022. Obes Pillars. (2022) 1:100010. doi: 10.1016/j.obpill.2022.100010, PMID: 37990703 PMC10662031

[32] Clément K Biebermann H Farooqi IS Van der Ploeg L Wolters B Poitou C. MC4R agonism promotes durable weight loss in patients with leptin receptor deficiency. Nat Med. (2018) 24:551–5. doi: 10.1038/s41591-018-0015-9, PMID: 29736023

[33] Clément K Van Den Akker E Argente J Bahm A Chung WK Connors H. Efficacy and safety of setmelanotide, an MC4R agonist, in individuals with severe obesity due to LEPR or POMC deficiency: single-arm, open-label, multicentre, phase 3 trials. Lancet Diabetes Endocrinol. (2020) 8:960–70. doi: 10.1016/S2213-8587(20)30364-8, PMID: 33137293

[34] Faccioli N Poitou C Clément K Dubern B. Current treatments for patients with genetic obesity. J Clin Res Pediatr Endocrin. (2023) 15(2):108–19. doi: 10.4274/jcrpe.galenos.2023.2023-3-2, PMID: 37191347 PMC10234057

